# Academic Integrity and Cheating in Dental Education: Prevalence, Drivers, and Career Implications

**DOI:** 10.3390/dj14010065

**Published:** 2026-01-19

**Authors:** Akhilesh Kasula, Gadeer Zahran, Undral Munkhsaikhan, Vivian Diaz, Michelle Walker, Candice Johnson, Kathryn Lefevers, Ammaar H. Abidi, Modar Kassan

**Affiliations:** College of Dental Medicine, Lincoln Memorial University, LMU Tower, 1705 St. Mary Street, Knoxville, TN 37917, USA; akhilesh.kasula@lmunet.edu (A.K.); gadeer.zahran@lmunet.edu (G.Z.); undral.munkhsaikhan@lmunet.edu (U.M.); vivian.diaz@lmunet.edu (V.D.); michelle.walker@lmunet.edu (M.W.); candice.johnson@lmunet.edu (C.J.); kathryn.lefevers@lmunet.edu (K.L.)

**Keywords:** integrity, cheating, dental school

## Abstract

**Background**: Integrity, encompassing honesty, accountability, and ethical conduct, is a cornerstone of the dental profession, essential for patient trust and safety. Despite its importance, academic dishonesty remains a pervasive issue in dental education globally. This review examines the prevalence, causes, and long-term career implications of academic dishonesty in dental education and explores institutional strategies to cultivate a culture of integrity. **Method**: The study was conducted using PubMed, Scopus, Web of Science, and Google Scholar to identify studies published between 1970 and 2025 on academic dishonesty in dental education. Search terms included dental students, cheating, plagiarism, and clinical falsification. Eligible studies reported prevalence, drivers, or consequences of dishonest behaviors. Data were extracted and thematically synthesized to highlight common patterns and professional implications. **Results**: Self-reported data indicate alarmingly high rates of cheating among dental students, ranging from 43% to over 90%. Common forms include exam fraud, plagiarism, and the falsification of clinical records. Key drivers include intense academic pressure, competitive environments, and a perception of weak enforcement. Such behaviors are not merely academic violations—they have profound professional consequences. A history of academic dishonesty can damage a student’s reputation, hinder licensure and credentialing processes, and limit postgraduate opportunities. Crucially, studies indicate that unethical behavior in school can normalize dishonesty, predicting a higher likelihood of future professional misconduct, such as insurance fraud or malpractice, thereby jeopardizing patient care and public trust. **Conclusions**: Academic integrity is a critical predictor of professional ethical conduct. Dental schools must move beyond punitive policies to implement proactive, multi-faceted approaches. This includes integrating comprehensive ethics curricula, fostering reflective practice, promoting faculty role modeling, and empowering student-led initiatives to uphold honor codes. Cultivating an unwavering culture of integrity is essential not only for academic success but for developing trustworthy practitioners committed to lifelong ethical patient care.

## 1. Introduction

Integrity refers to the quality of being honest and maintaining a steady and firm commitment to strong moral and ethical standards and values [1,2,3]. From an ethical standpoint, integrity is viewed as the honesty, truthfulness, and sincerity of an individual’s actions [2,4]. In dentistry, integrity serves as a foundational pillar that includes honesty, ethical accountability, and professionalism [2,3,4]. Integrity in dentistry is not limited to classroom instruction [2,5,6]. It embodies the principles that upcoming professionals are expected to uphold in patient interactions and professional relationships [2,7]. Core principles, such as trust, accountability, and respect for both patients and colleagues, form the basis of ethical dental practice [2,3]. Neglecting to instill these values can result in clinicians who are devoid of the moral foundation essential for responsible practice. Beyond shaping professional identity, integrity also forms the foundation of the social contract between health professionals and the communities they serve [4]. Dentistry, much like medicine, depends on the confidence patients place in their providers, and breaches of integrity during training can erode that trust over time [8,9]. For this reason, ethical standards in dental education are not simply internal guidelines but represent a commitment to accountability and responsibility to society at large.

Despite the emphasis on professionalism, academic dishonesty is a well-documented occurrence in dental education [10,11]. Dishonesty in this context includes examination fraud, plagiarism, falsification of clinical requirements, and misrepresentation of patient encounters [12,13,14,15,16]. Reported prevalence rates by students themselves are surprisingly high. Research indicates that between 43% and 94% of dental students have admitted to cheating at some point during their education [12,16,17,18,19,20]. A large-scale survey in South Korea revealed that 92.2% of students committed at least one form of academic dishonesty [12]. In the United States, dental school deans reported an average of one to two formal cases annually; however, they also noted that many incidents may remain unreported because of procedural difficulties [15]. These findings imply that although integrity is highly valued in dental education, violations may occur more often than institutional documentation suggests. Several systemic factors drive these behaviors, such as the pressure of competitive academic environments, the stress of high-stakes examinations, and cultural or institutional norms that may inadvertently allow misconduct to occur. Examining these drivers is valuable because they shed light on why dishonest practices continue despite well-defined professional expectations. In addition, looking at international contexts reveals how variations in educational systems and societal values can shape the patterns and prevalence of academic misconduct. Further supporting the influence of broader academic culture, Hanafi et al. reported that institutional norms, research culture, and systemic barriers within higher-education environments significantly shape students’ ethical decision-making and attitudes toward integrity [1].

Academic dishonesty does not involve just grades or disciplinary records. It significantly impacts professional identity and career development. Engaging in cheating behaviors threatens to normalize dishonesty and establish habits that could transfer into clinical practice [8,21,22]. In a profession where patient safety, informed consent, and accurate clinical documentation are crucial, dishonest behaviors will endanger patient outcomes and public trust [4]. Students who prioritize principles of integrity are more likely to have reputations of reliability and professionalism [8,23,24]. Because of these high stakes, integrity education should be approached not merely as adherence to rules, but as a developmental process that cultivates moral reasoning, resilience to peer influence, and the skills needed to manage ethical challenges in real clinical practice [25]. Viewed this way, integrity becomes both a personal virtue and a teachable competency that can be intentionally developed within dental curricula [7,26].

This review paper aims to examine the prevalence of unethical decisions in dental education, their consequences for both academic and professional development, and potential strategies to strengthen integrity. By integrating findings from U.S. and international studies, this review highlights the widespread nature of academic dishonesty and the need for systemic interventions. Ultimately, fostering ethical conduct in dental schools is not only a matter of academic policy but also a requirement for developing competent and trustworthy practitioners.

This review was conducted to synthesize existing evidence on the prevalence and consequences of academic dishonesty among dental students.

## 2. Methodology

A structured literature search was undertaken to ensure comprehensive coverage of the topic. Search was conducted across multiple electronic databases, including PubMed, Scopus, Web of Science, and Google Scholar, which together capture a wide range of biomedical, educational, and interdisciplinary research.

To enhance methodological rigor and reproducibility, the search strategy was expanded and aligned with PRISMA recommendations. The development of the search process involved several stages: identifying relevant keywords, mapping controlled vocabulary (MeSH, Emtree), and constructing database-specific search strings. Boolean operators (AND, OR), phrase searching, and truncation symbols were systematically applied to optimize both sensitivity and specificity. Keywords and phrases included: “dental students,” “academic dishonesty,” “cheating,” “plagiarism,” “exam fraud,” “clinical falsification,” and “professional misconduct.” For example, “cheat*” retrieved “cheating” and “cheaters”.

A representative full search string used in PubMed was: ((“dental students”[MeSH Terms] OR “dental student”[Title/Abstract] OR “dental education”[MeSH Terms]) AND (“cheating”[Title/Abstract] OR “academic dishonesty”[Title/Abstract] OR “plagiarism”[MeSH Terms] OR “clinical falsification”[Title/Abstract]) AND (“ethics”[MeSH Terms] OR “professional misconduct”[MeSH Terms])). Equivalent search strings were adapted for Scopus, Web of Science, and Google Scholar, incorporating filters for publication year, English language, and peer-review status.

The search was limited to English-language articles published between 1970 and 2025 to capture both historical and contemporary perspectives. This period was chosen because the earliest documented studies on dishonesty in dental education appeared in the late 1970s, and including literature through 2025 ensured the incorporation of emerging post-pandemic integrity research.

Reference lists of all included articles and relevant systematic or narrative reviews were manually screened to identify additional eligible studies not captured by database searches. Where full texts were not available online, institutional library services and interlibrary loans were used to retrieve them.

Studies were eligible for inclusion if they met the following criteria:

1. Empirical studies reporting prevalence, forms, or manifestations of academic dishonesty within dental education.
2. Studies examining determinants, motivations, or consequences of dishonest behaviors in dental students.
3. Peer-reviewed quantitative (e.g., cross-sectional surveys, longitudinal studies) or qualitative (e.g., interviews, focus groups) research.

Studies were excluded if they met any of the following conditions:

1. Opinion pieces, commentaries, or editorials lacking original empirical data.
2. Research conducted outside of dental education, unless findings from medical or allied health education provide directly comparable insights relevant to dental training.

To increase clarity and reproducibility, we expanded the study selection into a structured multi-stage process. First, two reviewers independently screened all titles and abstracts using the predefined inclusion and exclusion criteria. Articles that appeared relevant or potentially relevant were moved to full-text review. During the full-text assessment stage, the same reviewers independently evaluated each article for eligibility based on study design, population, relevance to dental education, and reporting of academic dishonesty outcomes. Any discrepancies between reviewers at either stage were resolved through discussion. If consensus could not be reached, a third reviewer adjudicated the decision to ensure consistency and reduce bias. The reasons for exclusion of full-text articles (e.g., non-empirical reports, wrong population, outside dental education, or lack of dishonesty outcomes) were documented and summarized in the diagram (Figure S1 and Tables S1–S3).

Data extraction was carried out using a standardized template capturing study characteristics, sample size, study design, type of academic dishonesty, prevalence data, and reported career or professional implications. Extracted information was synthesized using thematic analysis to identify recurring patterns across quantitative and qualitative studies. A PRISMA-aligned flow structure guided documentation of study identification, screening, exclusion, and final inclusion. By explicitly listing included studies in Table S1 and summarizing exclusion reasons in Table S3, the review provides a transparent and systematic account of the evidence base synthesized. To ensure transparency in how quantitative findings were reported, we clarified that all extracted numerical outcomes were considered during synthesis; however, when multiple studies reported overlapping variables, we highlighted those with larger samples, more recent publication dates, or unique contextual contributions (e.g., cross-cultural comparisons or distinctive forms of misconduct). Reported prevalence ranges (e.g., 43–94%) therefore reflect the full numerical span across all included studies, while studies cited explicitly in the manuscript were selected based on methodological strength, clarity of reporting, or their representativeness of broader trends. Other studies addressing similar outcomes were incorporated through grouped citations to maintain completeness and avoid selective reporting.

Quality assessment and risk of bias. To address variability in methodological rigor, all included empirical studies underwent a structured quality assessment. Two reviewers independently appraised each study using a checklist adapted from validated tools for cross-sectional and survey research. Five domains were evaluated: (1) sampling strategy and representativeness of the target population; (2) response rate and handling of non-response or missing data; (3) clarity and validity of instruments used to measure academic dishonesty and related constructs; (4) transparency and completeness of outcome reporting; and (5) consideration of contextual factors or potential confounders. Each domain was rated as low, moderate, or high risk of bias, with disagreements resolved by a third reviewer. In the narrative synthesis, studies judged to be of higher quality, based on more robust sampling, validated instruments, and complete reporting, were given greater interpretive weight. Summary quality ratings for all included studies are presented in Table S1.

Together, these revisions strengthen the transparency, comprehensiveness, and replicability of the review (Figure S1 and Table S1).

## 3. Results

To enhance clarity in interpreting quantitative findings, we note that the prevalence ranges and numerical estimates presented in this section reflect data from all included studies. Individual studies are highlighted when they offer particularly robust, recent, or contextually informative data; however, grouped references are used where multiple studies report similar outcomes to avoid redundancy and prevent selective emphasis.

### 3.1. The Foundations of Integrity in Dental Education

Integrity in dental education is based on well-established ethical frameworks that guide both the learning of students and their professional behavior. In the United States, the ADA Principles of Ethics and Code of Professional Conduct emphasize patient autonomy, nonmaleficence, beneficence, justice, and veracity [2]. Internationally, organizations such as the World Dental Federation (FDI) have issued guidelines emphasizing honesty, respect, and accountability as essential to dental practice [3]. At the institutional level, many dental schools have implemented honor codes and integrity policies that prohibit academic dishonesty while promoting a sense of professional responsibility [10,15]. These ethical frameworks serve as a bridge between the academic and professional environment, emphasizing that dishonesty in education violates the core values of dentistry.

Professionalism is a quantifiable element of dental education [11,27]. Key attributes such as accountability, honesty, and responsibility are essential for clinical competence and ensuring trust with patients (ADA and CODA). A student who falsifies patient interactions or clinical requirements may seem to fulfill graduation criteria on paper, but in reality, they lack the essential skills and habits required for safe practice [8,21]. Conversely, students who reliably show integrity in their academic and clinical duties are more likely to cultivate trust within professional relationships [4,7,13,14]. Studies in health professions education emphasize that the development of professional identity during training has lasting effects on clinical conduct [4,8,15,21]. Additionally, fostering professionalism within dental education serves as an ultimate goal and a protective measure for the welfare of patients.

An important inquiry within dental education is the extent to which ethical behavior in academic settings can predict future professional conduct [8,21,25]. Research indicates a positive correlation. Students who engage in academic dishonesty tend to justify unethical practices in their future careers [8,21,23,26,28]. Engaging in cheating can lead to the normalization of dishonest conduct, weaken moral judgment crucial for clinical decision-making, and diminish the perceived gravity of breaching ethical norms [22,29]. Conversely, emphasizing integrity as a fundamental competency throughout dental education strengthens ethical reasoning and accountability patterns that carry into the professional career.

### 3.2. Cheating in Dental Schools: Forms, Causes, and Prevalence

Academic dishonesty within dental education presents in several ways [10,16]. The most common forms include cheating, plagiarism, the fabrication of clinical records, and collusion [12,16,17]. Such actions are breaches of academic regulations and raise concerns regarding competence, patient safety, and professional preparedness.

Several factors play a role in the constant issue of cheating within dental education. Students often cite excessive stress, heavy workloads, and competitive environments as the main pressures that drive them towards dishonest behavior [28,30]. Some students justify misconduct by citing peer behaviors like “everyone does it” or by blaming weak enforcement of policies [12,31]. Cultural factors can also have an impact, as expectations regarding collaboration, academic pressure, and deference to authority differ across various contexts [23,32]. The combination of these pressures creates a place where academic shortcuts can be justified as necessary for survival, instead of being seen as violations of ethical standards. These themes were consistently observed across dental, medical, and pharmacy education studies from Korea, New Zealand, the Middle East, and India [12,13,17,32,33], which were selected for discussion in this section because they provide detailed analyses of context-specific drivers and are representative of broader patterns identified in the full set of included studies.

The prevalence of academic dishonesty in dental schools remains notably elevated according to various studies. Early survey work by Fuller, Killip, and Warman et al. documented substantial levels of cheating in U.S. dental schools [18,19], and one survey revealed that 43% of students admitted to cheating, whereas 94% noted that such behavior was common among their classmates [18,19]. Another study indicated that almost 30% of students recognize cheating as prevalent in dental education [23,30]. Recent surveys indicate even greater occurrence within dental programs in the U.S. and Canada. Between 57.5% and 74% of students admitted to engaging in various forms of academic dishonesty [12]. In dental hygiene programs, the reported rate was 86.5% [20,25]. These prevalence estimates are further supported by studies from Jordan, India, Malaysia, and Peru, which report comparable patterns of misconduct in different educational settings [16,17,27,32].

Dental institutions address occurrences of academic dishonesty through various disciplinary actions [9,31]. Depending on the severity of the offense, repercussions may include academic probation, remediation, or dismissal from the program. Many educational institutions implement honor codes, integrity pledges, and supervised examinations as preventive measures [5].

A historical comparison suggests that reported academic dishonesty has remained consistently high over time. Early studies in the 1970s–1990s reported prevalence rates ranging from 43% to 94%, as documented by Fuller & Killip (1979) [18] and Warman et al. (1994) [19]. Contemporary studies from 2007 to 2025 demonstrate similarly elevated or even higher rates, including 57.5–74% in U.S. and Canadian dental schools [23] and 92.2% in Korean dental students [12], as well as 86.5% among dental hygiene students [20]. While methodological differences across decades limit direct trend analysis, the persistence of high rates across time periods indicates that cheating in dental education is not a new phenomenon but an enduring challenge that continues despite institutional reforms and heightened awareness.

### 3.3. Career Consequences of Academic Dishonesty

Integrity serves as the foundation of a dentist’s professional reputation. Dishonest actions during training can compromise trust, not only among faculty and peers but also later among colleagues and patients [34]. Students who are caught cheating may encounter skepticism from faculty mentors, which could impact future recommendations and career opportunities [10,12]. In clinical practice, credibility and trust between a dentist and patient are crucial to their relationship. Once these elements are compromised, restoring them is very challenging. As Artioli (2011) conveys, professionalism and ethical conduct directly shape how practitioners are perceived, and early lapses of integrity in the career can have long-term reputational consequences [7].

Engaging in academic dishonesty can compromise the integrity of professional credentialing processes. Dental students found to have committed misconduct may face penalties that could delay or disqualify them from taking board examinations or obtaining licensure [26]. In instances of altered patient records or misrepresentation of clinical activities, students may be subject to accusations of fraud, which has both legal and professional consequences. Dental boards are especially aware of issues of character and ethics, indicating that unethical conduct recorded during training may emerge during the licensure evaluation [13]. Licensing boards explicitly evaluate applicants’ ethical conduct when determining eligibility for independent practice. State dental boards, such as California, Texas, and New York, require applicants to disclose academic misconduct, professionalism violations, or any institutional disciplinary actions [35,36,37]. These disclosures may delay licensure, trigger additional documentation requests, or, in cases involving falsified clinical records or fraudulent documentation, result in denial of licensure. The ADA’s Principles of Ethics and Code of Professional Conduct emphasize veracity and integrity as core obligations, and breaches of these standards during training raise direct concerns regarding applicant character. Longitudinal work by Papadakis et al. demonstrates that professionalism lapses during training are associated with later licensure board disciplinary actions, supporting the regulatory rationale for close scrutiny [8]. Consequently, academic dishonesty is not only an institutional concern but also an issue with clear downstream implications for credentialing, licensure approval, and long-term regulatory oversight.

Engaging in cheating can also significantly affect postgraduate opportunities. Instances of academic dishonesty may lead to disciplinary marks on a student’s educational record, consequently affecting admission to residency programs, academic roles, or specialized training. Many programs require faculty recommendations and academic assessments that evaluate intellectual ability, professionalism, and integrity [12,38]. A history of dishonesty negatively affects these assessments and can generalize candidates as high-risk individuals, thereby limiting their career paths compared to colleagues with flawless records.

One of the most significant concerns is the potential that dishonest behaviors committed during dental school are associated with a higher likelihood of future professional misconduct, rather than demonstrating a direct causal progression. Studies in medical and dental education indicate that dishonesty can lead to the normalization of unethical practices [10,23,33]. In particular, Papadakis et al. and related longitudinal work in medical education have shown that professionalism concerns documented during training predict subsequent disciplinary action by medical boards [8,21]. These studies were therefore highlighted in this section as exemplary evidence linking academic integrity to later clinical behavior. These studies indicate that early ethical lapses correlate with, but do not prove, later violations in clinical practices. Students who justify plagiarism or falsified records are more likely to justify insurance fraud, malpractice, or other ethical violations in dental practice [12,21,22]. Conversely, institutions that enforce rigorous academic integrity standards strengthen the principle that honesty is a fundamental component of competent patient care. Integrity functions as both a safeguard for patients and an indicator of ethical behavior in professional practice [8].

When self-reported prevalence data from published studies are applied to a typical dental-education cohort, the magnitude of academic dishonesty becomes clear. For example, Fuller & Killip (1979) found that 43% of U.S. dental students admitted to cheating, while 94% believed that cheating was common among their peers [18]. Similarly, Muhney et al. (2008) reported that 86.5% of graduating dental-hygiene students acknowledged at least one act of academic dishonesty [20]. Although other studies (e.g., Warman et al., 1994; Andrews et al., 2007) examined attitudes and perceptions rather than direct admission rates [19,23]. The combined evidence indicates that academic misconduct occurs at levels substantial enough to affect student progression.

Based on these empirically supported ranges (43–86.5%), a class of 100 students could realistically include 40–85 individuals who have engaged in at least one dishonest behavior. Even if only a fraction of these incidents are formally adjudicated, institutions may still see 5–15% of a cohort placed on probation, remediation, or dismissed for severe violations, rates high enough to influence CODA-monitored attrition metrics and to carry long-term implications for licensure, residency placement, and professional reputation.

### 3.4. Institutional Strategies to Uphold Integrity

The best way to prevent academic dishonesty is the enforcement of explicit policies and honor codes. Most dental schools in the United States and Canada maintain integrity policies, and the implementation of honor codes or pledges that clearly outline the expectations for ethical behavior [10,15].

In addition to policies, preventive measures are crucial for eliminating opportunities for cheating. Common strategies include monitored examinations, rotation of questions, and the use of plagiarism detection software, all of which disrupt academic dishonesty [6,13]. Supervised examinations, whether conducted in person or online, significantly decrease the chances of dishonest behavior. These institutional safeguards send a clear message that academic misconduct is unacceptable, thereby preserving the integrity of testing results. By reducing temptation and opportunity, these preventive strategies enhance broader ethical training initiatives.

Dental schools must foster a culture of integrity by means of faculty role modeling and mentorship. Students frequently indicate that witnessing professional behavior in their educators significantly influences their own ethical decision-making [12].

When students believe that faculty overlook misconduct or do not exemplify the values they advocate, dishonesty tends to become normalized [39]. Additionally, peer accountability is critical, as students are less likely to engage in cheating when integrity is collectively upheld within their group [23]. Ultimately, educational institutions can eliminate one of the main contributors to dishonesty and stress by decreasing workload to include high-yield topics and to not be busy work, removing unnecessary electives, providing wellness resources, time management tools, and encouraging meetings between students and faculty. Together, these approaches emphasize that integrity is beyond rule following; it symbolizes a collective dedication to professionalism and the safety of patients.

By mitigating academic dishonesty, institutions also reduce the likelihood of disciplinary actions such as probation or dismissal, which can disproportionately elevate attrition rates beyond CODA’s expected benchmarks (~7%) [31]. The consistent enforcement of clear integrity policies, combined with preventive safeguards such as secure examination protocols and plagiarism detection, reinforces accountability and fairness. Equally important is the visible commitment of faculty to ethical role modeling and mentorship, which strengthens the professional culture of the institution. Together, these measures not only preserve academic integrity but also safeguard student retention, ensuring that dental graduates embody the standards of professionalism essential for maintaining public trust and delivering safe patient care [9].

### 3.5. Cultivating Ethical Future Dentists: Proactive Approaches

One of the most effective methods to promote integrity within dental education is through structured ethics curricula that extend beyond policy adherence [6,26,40]. By incorporating case-based discussions, workshops, and practical scenarios, students are tasked with opportunities to display moral reasoning and ethical decision-making actively [33]. One of the most effective approaches is not to treat ethics as just a theoretical construct; instead, these exercises connect professional values directly to clinical practice. By analyzing scenarios like reconciling patient autonomy with treatment recommendations, students can hone their ability to implement ethical reasoning in actual clinical situations [2,4,8].

The effectiveness of ethics education is improved when it is combined with opportunities for reflection and self-evaluation [24]. Motivating students to investigate their own decision-making processes, biases, and professional obstacles creates a stronger sense of personal responsibility [23].

Promoting a culture of integrity demands the empowerment of students. Peer-led initiatives, such as honor pledges, ethics clubs, or peer mentoring programs, enable students to hold each other accountable while simultaneously reinforcing the values of the community [31,41]. Campaigns focused on student engagement regarding academic integrity and professional ethics can help reduce the stigma associated with reporting misconduct, while also encouraging discussions about the pressures that lead to cheating [30]. When students are involved in defining their professional environment, the concept of integrity transforms into a collective obligation instead of a directive enforced by higher authorities.

Ultimately, strengthening professionalism in dental education requires proactive approaches that integrate ethics into both the curriculum and clinical practice [5]. Developing transparent evaluation systems, open communication regarding grading and clinical expectations, and consistent feedback mechanisms reduces ambiguity that often fosters ethical lapses [34]. Embedding ethical reasoning into routine patient care through case-based simulations, reflective exercises, and transparent clinical decision-making ensures that students recognize integrity as inseparable from competent practice [38]. When paired with faculty mentorship and peer-driven initiatives, these strategies not only prevent misconduct but also create a culture of trust and accountability. By aligning academic policies, curricular design, and clinical training with a commitment to transparency, dental schools can prepare graduates who embody the highest ethical standards, safeguarding patient welfare and reinforcing public confidence in the profession.

Across dental-related studies, reported prevalence of academic dishonesty ranged from 30% to more than 92%, with rates particularly high in dental hygiene programs (86.5%) and international dental schools (up to 92.2%). Common drivers included stress, competitive environments, unclear policies, peer norms, and weak institutional enforcement. These behaviors were consistently linked to diminished professional identity formation, reduced moral competence, and increased risk of unethical conduct in clinical practice, highlighting the need for robust institutional strategies such as honor codes, clear policy communication, strengthened supervision, and integrated ethics education (Table 1).

Collectively, the evidence demonstrates that academic dishonesty is not an isolated student issue but a systemic challenge affecting institutional culture and patient safety. High-stress, high-stakes learning environments foster unethical behaviors unless paired with strong ethical frameworks and faculty role modeling. Institutions with transparent grading, faculty professionalism, structured ethics curricula, and consistent enforcement report lower prevalence of misconduct. Quality-assessment findings highlight variable study rigor, reinforcing the need for standardized global reporting systems. Policy implications call for integrated ethics training from year one, improved communication channels for reporting misconduct, and formative assessment systems that reward honesty and professionalism. Ultimately, integrity must be treated as a clinical competency equivalent in importance to technical skill.

## 4. Discussion

The findings of this review demonstrate that integrity in dental education is deeply rooted in established ethical frameworks, yet consistently challenged by the pressures and realities of modern academic environments. Professional guidelines such as the ADA Principles of Ethics and Code of Professional Conduct [39] and the FDI’s global policy on dental ethics [3] standards stress honesty, accountability, patient autonomy, and veracity as foundational expectations for dental practitioners. However, the results clearly show that academic dishonesty remains pervasive across dental institutions worldwide, with reported cheating rates ranging from 43% to 94% [12,18,19,20] in student populations and even higher in dental hygiene programs [20]. These behaviors emerge in multiple forms, including plagiarism, exam cheating, falsification of clinical records, and inappropriate collaboration [23,29,32,42], and are driven by a complex interplay of excessive academic workload, competitive learning environments [12,15,23,32], inadequate faculty oversight, and cultural norms that may unintentionally normalize unethical shortcuts [12,13]. The data highlight that when such misconduct occurs, the consequences extend far beyond academic performance: they fundamentally erode professional identity formation [8,11], compromise moral reasoning [25,26,43], and weaken the ethical judgment essential for safe and responsible dental practice [7,22].

Importantly, the results highlight a strong association between unethical behavior during dental school and future misconduct in clinical practice. Students who justify dishonest academic behaviors are more likely to engage in similar patterns of unethical reasoning in clinical settings, rationalize fraudulent documentation, malpractice, and violations of professional standards once they enter the workforce. This relationship reflects an association rather than a proven causal pathway [8,21]. This pattern presents significant risks: compromised patient trust [4,34], diminished public confidence in the profession [9], and potential legal ramifications [8,15]. Furthermore, documented incidents of dishonesty can jeopardize students’ opportunities for licensure, postgraduate training, and professional advancement, as many licensing bodies and residency programs place high value on professionalism and character [8,35,36,37] because direct longitudinal data in dental education remain limited, these findings should be interpreted as correlational and not causal. Institutions, therefore, play a decisive role in shaping ethical development; schools with clear honor codes, consistent enforcement policies, secure examination systems, and strong faculty role modeling report lower rates of misconduct [10,23,30]. Conversely, environments where policies are inconsistently applied or where faculty fail to exemplify ethical conduct contribute to a culture in which dishonesty becomes normalized [12]. This is particularly concerning given that even a small proportion of students facing probation or dismissal for misconduct can significantly elevate attrition rates beyond acceptable CODA thresholds [31].

Collectively, the results make evident that fostering a culture of integrity requires proactive, system-wide educational strategies rather than reliance on punitive measures alone. Ethics curricula that incorporate case-based scenarios, reflective practice, and guided discussions help students connect moral reasoning to real clinical decision-making [4,6,43]. Peer-led initiatives, mentoring programs, and transparent evaluation processes further reinforce a shared sense of accountability [10,41]. Moreover, reducing unnecessary academic burdens, improving faculty-student communication, and providing wellness and time-management support can directly mitigate stress-based drivers of dishonesty [5,33,34]. Additionally, contemporary challenges such as digital misinformation further highlight the importance of information integrity, as Alhomsi et al. demonstrated that public interactions with dental content on social media can amplify inaccuracies and erode trust when professionals do not model responsible communication practices [44]. These findings are consistent with broader higher-education literature demonstrating that cheating and plagiarism are pervasive and multifactorial challenges across university settings [45].

Taken together, these findings point to a broader institutional responsibility: ensuring that integrity is taught, reinforced, and evaluated with the same rigor as clinical competencies [4,7,11]. If academic dishonesty continues unaddressed, the implications extend beyond educational environments, ultimately compromising patient safety and public trust [4,5,9,34]. Strengthening ethical culture in dental schools is therefore essential not only for upholding academic standards but for shaping future clinicians who embody the professionalism and moral responsibility foundational to the dental profession [4,7,38].

The mechanisms outlined in Table 2 further reinforce that academic dishonesty in dental education is not the result of isolated student behavior but arises from a convergence of individual pressures, interpersonal influences, and institutional gaps. As shown, high-stakes testing environments, competitive grading systems, and overwhelming workloads frequently interact with peer norms and weak policy enforcement to normalize dishonest practices. These mechanisms directly threaten the development of professional identity, as students exposed to permissive or inconsistent integrity cultures are more likely to carry unethical habits into clinical decision-making, documentation, and patient management. The professionalism and licensure-related consequences mapped in the table highlight that early lapses, particularly those involving falsified clinical records or misrepresentation of patient encounters, have long-lasting effects on credibility, trust, and postgraduate opportunities. Importantly, the institutional implications highlight the need for comprehensive reform: schools must strengthen policy clarity, enforce consequences consistently, model ethical behavior through faculty conduct, and redesign curricula to reduce unnecessary stressors that fuel misconduct. Integrating these systemic insights into educational planning is essential to prevent a cyclical pattern in which academic dishonesty evolves into professional misconduct, ultimately compromising patient safety and public trust.

### Limitation

This review has several limitations that warrant acknowledgment. Most included studies relied on self-reported data, introducing possible recall and social-desirability biases. Publication bias may also be present, as institutions with lower rates of academic dishonesty may be less likely to publish such findings. Considerable heterogeneity across educational systems, countries, survey instruments, and definitions of misconduct limits direct comparison of prevalence estimates. Additionally, this review included only peer-reviewed, English-language publications, excluding grey literature and non-English evidence. Finally, inconsistent reporting across studies made meta-analysis infeasible. These limitations should be considered when interpreting the findings and their implications for dental education.

## 5. Conclusions

Integrity is a fundamental principle of dental education and professional practice, serving as the foundation for trust, patient safety, and career success. As discussed in this paper, academic dishonesty in dental schools manifests in various ways, from cheating on exams to the falsification of clinical records, and is often driven by stress, competition, and cultural factors. While engaging in academic misconduct may offer temporary comfort, it causes significant long-term repercussions. Dishonesty undermines professional credibility, jeopardizes licensure, and can ultimately endanger patient care.

The message is clear: engaging in cheating leads to lasting damage to one’s integrity. Academic dishonesty is not limited to the educational environment, as tendencies towards unethical conduct frequently accompany students into their clinical careers, where the consequences are significantly more severe. Academic dishonesty threatens the integrity of dental education, undermines public trust, and predicts future ethical failures in clinical practice. This comprehensive review demonstrates that dishonesty is prevalent, multifactorial, and deeply intertwined with institutional culture. Dental schools must implement systemic approaches, including ethics curricula, transparent evaluation, mentorship, wellness programs, and consistent policy enforcement, to safeguard educational quality. Future practitioners must be trained not only in clinical excellence but in unwavering integrity to ensure patient safety and long-term professional credibility.

For these reasons, dental schools must be on top of their dedication to creating an ethical culture, one that incorporates systematic ethics education, preventive measures, and mentorship throughout all phases of training. By establishing environments that value honesty and professionalism alongside technical proficiency, institutions safeguard their integrity while helping students to become competent practitioners. The future of dentistry relies on graduates who exemplify integrity, ensuring that both expertise and ethical accountability dictate patient care.

## Figures and Tables

**Table 1:** Summary of empirical findings from dental-related studies examining academic dishonesty, including prevalence rates, identified drivers, reported effects on professionalism and career development, and institutional responses. This table synthesizes data exclusively from studies conducted within dental schools or dental hygiene programs, removing all non-dental health-education literature. Prevalence values reflect self-reported or observed misconduct rates where available, while qualitative studies are summarized by thematic findings. Studies highlighted include early foundational surveys (1979–1994), large multi-institutional investigations (U.S., Canada, Republic of Korea, India, Peru), and recent analyses of moral competence and professionalism in dental learners. The table provides a comparative overview of misconduct patterns, contributing factors, and institutional gaps across different educational settings.

Reference	Prevalence of Cheating/Misconduct	Primary Drivers Identified	Professionalism/Career Outcomes	Institutional Responses/Policies
[18]	43% admitted cheating; 94% believed peers cheated	High workload; peer norms (“everyone does it”)	Early normalization of dishonesty	Weak enforcement; lack of clear definitions
[19]	~30% acknowledged cheating; widespread perception of dishonesty	Stress; competitive environment	Credibility issues with faculty; trust erosion	Limited documentation; informal handling
[20]	86.5% committed ≥1 dishonest act	Prior cheating; peer influence	Predictor of low professional integrity	Need for structured ethics curriculum
[23]	57.5–74% admitted misconduct across categories	Weak policy clarity; hidden curriculum; peer norms	Reduced trust between faculty & students	Honor codes inconsistently enforced
[12]	92.2% committed ≥1 dishonest behavior	Cultural norms; exam stress; pressure to perform	Risk of long-term tolerance of unethical conduct	Stronger supervision and policy clarity recommended
[15]	Policy study; no prevalence data	Variability in enforcement	Inconsistent faculty modeling undermines values	Wide variability in U.S. dental school policies
[32]	Misconduct frequently observed by faculty	Stress; unclear expectations; inequities	Weak moral-competence development	Standardized policy development needed
[42]	High plagiarism prevalence (exact % not reported)	Digital access; unclear plagiarism norms	Weak academic-ethics foundation	Need for digital-ethics instruction
[16]	Cheating reported as common (no % but high frequency)	Poor proctoring; opportunity; peer behavior	Ethical desensitization	Recommended stronger proctoring & monitoring
[17]	High prevalence across six dental colleges (“common practice”)	Peer norms; unclear penalties	Undermining of professional values	Student–faculty perception gap highlighted
[13]	Moderate–high dishonesty rates (varied: 40–70% depending on behavior)	Cultural expectations; low reporting; stress	Erosion of trust culture	Advocates trust-building & clarity of policies
[11]	Not prevalence; focused on moral competence	Low ethical reasoning ability	Poor professional performance trajectory	Need for structured moral-competence training
[7]	Not prevalence; conceptual professional ethics	Weak ethical identity	Long-term reputational risk	Dental ethics integration required
[24]	Not prevalence; focuses on professionalism	Weak professionalism training	Threat to licensure, patient trust	Advocates stronger training in professionalism

**Table 2:** Mechanisms and drivers of academic dishonesty in dental and health-professions education, with associated implications for professionalism, career development, and institutional policy. This table summarizes key behavioral, environmental, and systemic factors that contribute to academic dishonesty across dental and closely related health-professions programs. Mechanisms are categorized into individual, interpersonal, and institutional drivers, with corresponding impacts on ethical development, clinical professionalism, licensure pathways, and patient-care readiness. Institutional implications highlight gaps in policy enforcement, the role of faculty modeling, and vulnerabilities in assessment systems. Together, these data illustrate how academic misconduct emerges from a multifactorial interplay between personal pressures, cultural norms, and educational structures.

Mechanism/Driver	How it Promotes Academic Dishonesty	Representative Evidence (Dental/Health-Professions)	Professionalism/Career Implications	Institutional Levers/Responses
**Lack of safe reporting mechanisms**	Students may witness cheating but avoid reporting due to fear of retaliation, social isolation, or belief that “nothing will happen”	Refs. [13,41] emphasize fear and distrust as barriers to reporting academic misconduct	Silence in the face of misconduct can become a habit, later affecting willingness to report unsafe care, impaired colleagues, or fraud.	Anonymous reporting tools, anti-retaliation policies, faculty champions for integrity, and feedback to students on how reports are handled.
**Digital environment and information ecosystems**	Easy access to online materials, group chats, and contract cheating platforms lowers the barrier to plagiarism and answer-sharing; exposure to misinformation can blur lines for acceptable practice.	Ref. [44] (Interaction with dental misinformation online) highlight digital pressures and weak digital literacy.	Poor digital professionalism may extend to online patient communication, marketing, and representation of clinical outcomes.	Digital-ethics training (use of AI, plagiarism, social media), clear guidelines on online conduct, and use of plagiarism-detection and exam-security technologies.
**Documented professionalism concerns predicting later sanctions**	Repeated unaddressed lapses in professionalism during training signal risk for future regulatory problems	Ref. [8] shows that students with professionalism citations are significantly more likely to face later board disciplinary action.	Direct evidence that “small” lapses in training can translate into serious career consequences (License restriction, board action)	Systemic documentation of professionalism issues, structured remediation plans, and using integrity metrics as part of progression/advancement decisions.
**High academic stress and workload**	Heavy course load, dense exams, and perceived impossibility of success push students toward shortcuts (copying, unauthorized collaboration, exam cheating) as “survival strategies.”	Dental and health-professions students consistently cite stress, workload, and high-stakes exams as primary reasons for cheating (e.g., [12,17,32,33].	Habitual use of shortcuts under pressure can normalize rule-bending and undermine resilience when facing stressful clinical situations.	Rationalize assessment design (focus on high-yield content), reduce “busy work,” distribute workload more evenly, and integrate wellness and time-management support.
**Perceived peer norms (“everyone does it”)**	When students believe most classmates cheat, dishonesty becomes socially acceptable; fear of being at a “competitive disadvantage” overrides personal values.	Ref. [18] (43% self-reported cheating, 94% perceived peer cheating); Refs. [23,28] highlight peer norms and perceived prevalence as central drivers.	Aligns students with group norms rather than professional codes; can carry into practice as tolerance for “what everyone does” (upcoding, cutting corners).	Visible enforcement of policies, peer-led honor systems, ethics clubs, and campaigns that reshape norms around integrity and reporting.
**Ambiguous or weakly enforced policies**	Unclear definitions of cheating or inconsistent consequences lead students to view rules as negotiable or symbolic rather than binding.	Refs. [15,23] show that many schools have policies but uneven communication and enforcement; students often are unsure what counts as misconduct.	Early experience that rules are not applied consistently may generalize to future disregard of regulations (documentation, billing, infection control).	Clear, accessible policies; orientation sessions; consistent documentation of violations; faculty training to reduce variability in responses.
**Opportunity and low perceived risk of detection**	Poorly proctored exams, recycled questions, and undetectable copying create a high-reward, low-risk environment for cheating.	Refs. [10,16,17] describe frequent classroom and exam-related misconduct where surveillance is weak.	Encourages a mindset that unethical behavior is acceptable if one can “get away with it,” undermining internalized professionalism.	Secure assessment platforms, proctoring, randomized questions, plagiarism-detection tools, and audit trails for clinical documentation.
**Cultural and contextual norms**	In some contexts, sharing answers or “helping friends” is seen as loyalty rather than misconduct; hierarchies may discourage reporting.	Refs. [12,13,32] describe culture-specific attitudes that shape what behaviors are considered cheating or reportable.	If unaddressed, students may carry culture-specific rationalizations to global practice environments where standards differ, leading to conflicts with licensing and regulatory expectations.	Culturally sensitive ethics teaching, explicit discussion of global professional standards, safe/anonymous reporting channels, and faculty role-modeling of how to handle misconduct.
**Prior cheating history and learned rationalizations**	Students who have cheated previously (school or college) are more likely to repeat dishonesty and to develop justifying narratives (“no one is hurt,” “grades don’t reflect real ability”).	Ref. [20] identifies prior dishonest behavior as a predictor of current academic dishonesty; Ref. [21] show “carryover” from academic dishonesty to workplace misconduct.	Entrenched rationalizations can later support insurance fraud, falsifying records, or misrepresentation of outcomes in clinical practice.	Early identification and remediation of dishonesty, reflective exercises on past behavior, and longitudinal professionalism mentoring.
**Low moral competence/weak ethical reasoning**	Students who struggle to recognize ethical dimensions or weigh consequences are more prone to rationalize or minimize cheating.	Refs. [11,25,26,29] show links between lower moral competence/ethical sensitivity and higher tolerance or incidence of dishonest acts.	Weak moral reasoning in school predicts vulnerability to unethical decisions in complex clinical scenarios (e.g., consent, financial conflicts, over-treatment).	Longitudinal ethics curricula, case-based discussions, structured reflection, and explicit assessment/feedback on moral reasoning and professional judgment.
**Underdeveloped professional identity**	When students see themselves primarily as “test-takers” rather than future clinicians, they view cheating as an academic issue, not a professional breach.	Refs. [4,11,40] highlight that weak professional identity correlates with tolerance of unprofessional behaviors.	Delays the internalization of obligations to patients and society; increases risk that dishonest habits persist into clinical practice.	Early, explicit professional-identity formation (white-coat ceremonies, mentorship, patient contact), linking classroom behavior to future patient safety and trust.
**Faculty modeling and hidden curriculum**	If faculty cut corners, ignore misconduct, or send mixed messages, students learn that rules are negotiable despite official policies.	Refs. [10,23] describe discrepancies between formal policies and what students see faculty tolerate or do in practice.	“Hidden curriculum” can override formal ethics teaching, leading students to emulate unethical patterns they observe in authority figures.	Faculty development on role-modeling, explicit expectations in evaluations, recognition for faculty who exemplify integrity, and addressing faculty misconduct transparently.
**Assessment design and grading culture**	Overemphasis on high-stakes, norm-referenced exams and opaque grading fosters competition and grade obsession, which encourages cheating.	Refs. [5,30,33] link competitive, high-stakes assessment with increased likelihood of misconduct.	Encourages performance-orientation rather than mastery or patient-centered learning, eroding intrinsic ethical motivation.	Diversify assessment (formative OSCEs, reflective assignments), increase transparency, and align grading with demonstrated competence and professionalism.

## Data Availability

No new data were created or analyzed in this study. Data sharing is not applicable to this article.

## Supplementary Materials

The following supporting information can be downloaded at: https://www.mdpi.com/article/10.3390/dj14010065/s1, Figure S1: Summary of the Methodological Framework Used in the Systematic Review; Table S1: Full Database Search Strategy Used in This Review.; Table S2: Characteristics of the 37 Studies Included dishonesty and integrity in Final Synthesis; Table S3: Representative List of Excluded Studies with Corresponding Reasons for Exclusion.
