# Supplementary material for: Academic Integrity and Cheating in Dental Education: Prevalence, Drivers, and Career Implications
Source: Dent J (Basel). 2026 Jan 19;14(1):65. doi: 10.3390/dj14010065 (PMC12839645; doi:10.3390/dj14010065)
Supplement: Supplementary file 1 [file dentistry-14-00065-s001.zip › dentistry-3928075-supplementary.pdf]

dentistry journal

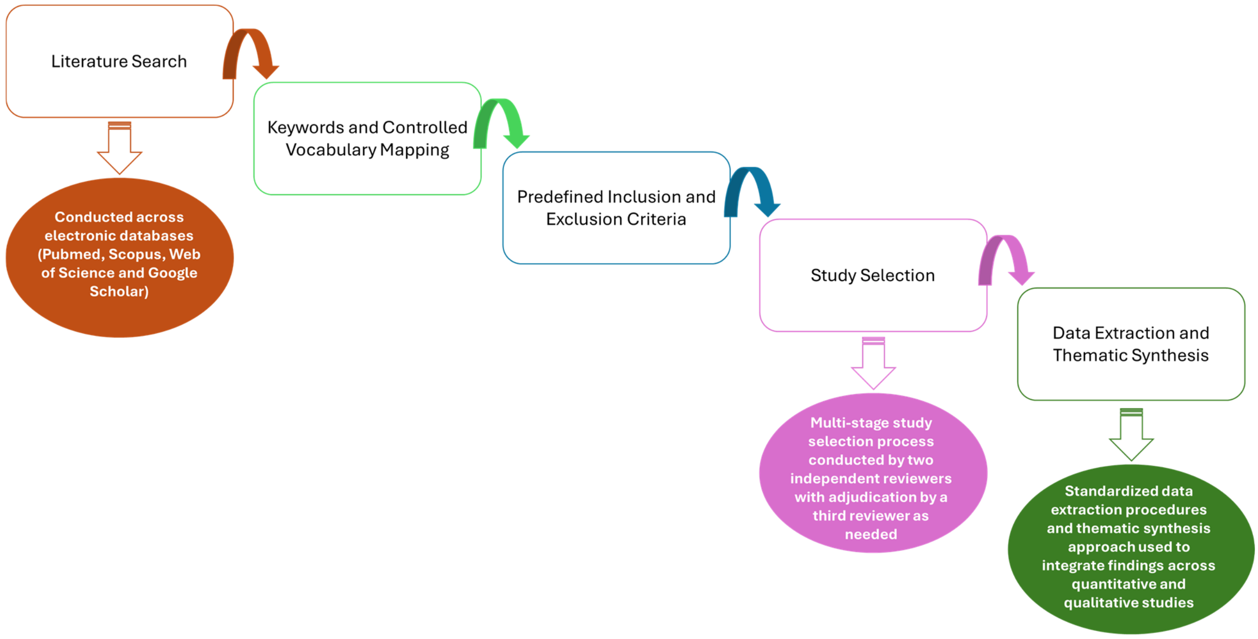

Figure S1. Summary of the Methodological Framework Used in the Systematic Review

Table S1. Full Database Search Strategy Used in This Review.

Database	Search String Used	Filters Applied	Notes
**PubMed (1970-2025)**	("Dental Students"[MeSH] OR "Students, Dental"[MeSH] OR dental student[tiab] OR "dental hygiene student"[tiab] OR "predoctoral dental"[tiab] OR "dental education"[tiab] OR "health profession student"[tiab] OR "clinical trainee"[tiab]) AND ("Academic Dishonesty"[MeSH] OR academic dishonesty[tiab] OR cheating[tiab] OR cheat[tiab] OR plagiarism[tiab] OR misconduct[tiab] OR "professional misconduct"[MeSH] OR "clinical falsification"[tiab] OR "assessment fraud"[tiab] OR "ethical sensitivity"[tiab] OR "ethical reasoning"[tiab] OR "moral competence"[tiab]) AND (education[tiab] OR curriculum[tiab] OR assessment[tiab] OR "clinical education"[tiab] OR "competency-based education"[tiab])	Language: English	
**Scopus (1970-2025)**	TS=(dental student OR "dental education" OR "dental hygiene student" OR "health profession student" OR "clinical trainee") AND TS=("academic dishonesty" OR cheating OR plagiarism OR misconduct OR falsification OR "professional misconduct" OR professionalism OR "ethical reasoning" OR "moral competence") AND TS=(education OR curriculum OR assessment OR "clinical education")	Language: English	
**Web of Science (Core Collection 1970-2025)**	"cheating in dental school"		Document type: Original Article
**Google Scholar (1970-2025)**	"dental students" "academic dishonesty"; "cheating in dental school" plagiarism; "dental education ethics" professionalism; "health profession students" "academic misconduct"; "moral competence" dental	Used for citation chaining, forward/backward searches, and uncovering grey literature.	

Table S2. Characteristics of the 37 Studies Included dishonesty and integrity in Final Synthesis.

Study	Population	Design	Core Focus
[18]	Dental students	Survey	Early prevalence of cheating
[19]	Dental students	Survey	Cheating typologies
[15]	Faculty	Policy survey	Integrity policies
[16]	Dental students	Survey	Cheating behaviors
[8]	Physicians	Cohort	Misconduct in training → Board action
[26]	Dental schools	Qualitative	Gaps in ethics curricula
[5]	Dental education	Narrative empirical	Professionalism decline
[20]	Dental hygiene	Survey	Predictors of dishonesty
[10]	Dental students	Survey	Integrity status/community norms
[12]	Dental students	Survey	Factors associated with cheating
[9]	Dentists	Media analysis	Professional misconduct
[28]	Univ students	Review	Why students cheat
[29]	Nursing	Survey	Dishonesty profiles
[22]	Dental faculty	Regression	Misconduct perceptions
[13]	Health-professions	Survey	Integrity perceptions
[31]	University	Intervention	Preventing misconduct
[45]	HEI students	Review	Cheating & plagiarism
[29]	Nursing	Survey	Moral competence & dishonesty
[27]	Dental	Survey	Professionalism lapses
[23]	Faculty/students	Survey	Misconduct perceptions
[43]	Health-professions	Longitudinal	Ethics competence
[11]	Dental	Mixed methods	Moral competence
[40]	Dental	Qualitative	Unprofessionalism experiences
[22]	Adults	Experimental	Dishonesty escalation
[42]	Dental	Survey	Perception of plagiarism
[17]	Dental	Survey	Student/faculty attitudes
[30]	University	Survey	Motivation & norms
[33]	Medical/pharmacy	Cross-sectional	Dishonesty & ethics
[25]	Dental	Psychometric	Ethical sensitivity
[41]	Students	Qualitative	Understanding integrity
[24]	Dental	Review	Professionalism
[14]	Nursing	Qualitative	Trust building
[7]	Dental	Review	Professional ethics
[34]	Ethics	Conceptual	Moral duty to stakeholders
[4]	Leadership	Conceptual	Integrity framework
[2]	Dental ethics	Guideline	Code of conduct
[3]	Dentists	Policy	Ethics statement

Table S3. Representative List of Excluded Studies with Corresponding Reasons for Exclusion.

Study	Exclusion Reason
Editorial on cheating (no data)	Not empirical
Letter to the editor on ethics	No data
High-school cheating study	Not comparable population
Study on business student ethics	Not health-profession relevant
Psychological constructs, no education context	Out of scope
Non-retrievable article (3 attempts)	Full text unavailable
Ethics simulation in engineering	Not comparable
Medical ethics lecture reflection	No integrity behavior outcomes
Commentary on "professionalism crisis"	No data
Veterinary student professional identity	Context differs significantly
Paper on bioethics philosophy	Not empirical
Evaluation of hospital administrators	Not learner-based
Nursing clinical ethics without misconduct	Outcomes not aligned
Pharmacy dispensing errors study	Not academic dishonesty
Obstetrics ethics case series	Not student population
Internal policy memo	Non-peer-reviewed
Undergraduate cheating in general population	No health-profession context
Sociology major cheating	Not comparable
Religious studies ethics	Not educational misconduct
Dental patient ethics viewpoint	Not student behavior
Internship professionalism rubric	No misconduct outcomes
Non-English full text unavailable	Failed retrieval
Data duplicate from another included study	Redundant
Meta-analysis without primary data	Excluded as per criteria
Conference abstract	Insufficient data
